# High-Areal-Loading Zinc-Ion Batteries with Long-Term Cycling at Practical Current Densities with Scalable Electrode Design

**DOI:** 10.1021/acs.nanolett.5c03864

**Published:** 2025-09-29

**Authors:** Md Zahidul Islam, Choongho Yu

**Affiliations:** † Department of Mechanical Engineering, 2655 Texas A&M University, College Station, Texas 77843, United States; ‡ Department of Materials Science and Engineering, Texas A&M University, College Station, Texas 77843, United States

**Keywords:** aqueous, carbon nanotube, stationary energy storage, low C rate, dry electrode

## Abstract

Aqueous zinc-ion batteries (ZIBs) offer a safe and cost-effective solution for stationary energy storage, but achieving long-term cycling at high areal loadings and low C-rates remains challenging. Here, we present a polyaniline-based ZIB with a unique 3D interconnected sponge-like carbon nanotube (CNT) host that provides high porosity, mechanical resilience, and robust conductivity. This architecture supports active material loading up to 6 mg cm^–2^, enabling stable cycling at practical current densities. With a dimethyl sulfoxide electrolyte additive, the cell retains 70% capacity over ∼6,000 cycles at 0.68C, highlighting its outstanding long-term stability at low rates. It also maintains ∼9,000 cycles at 6.8C, demonstrating high-rate capability. To demonstrate scalability, we implemented a solvent-free dry electrode process using CNT chunks and polytetrafluoroethylene binder, achieving a high areal loading of 7.9 mg cm^–2^ and delivering 140 mAh g^–1^ at 0.5 C. These results represent a significant step toward durable, high-loading, and scalable ZIBs for grid-level energy storage applications.

## Graphical abstract

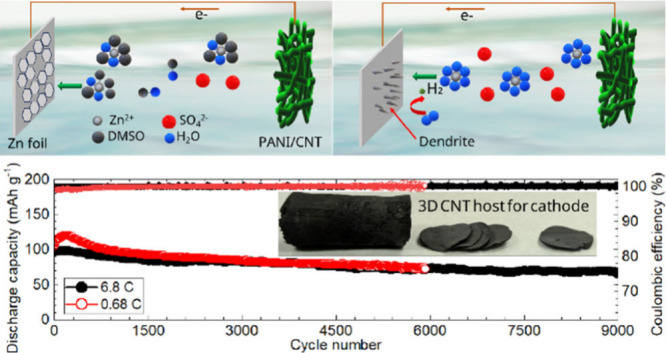

Global warming has risen by 1.0 °C due to human activity and could reach 1.5 °C between 2030 and 2052 if emissions persist.^1^ Renewable energy is key to addressing this issue; however, advanced batteries are required due to their intermittent nature.^2^ While lithium-ion batteries (LIBs) dominate the market, their flammable electrolytes and scarce lithium resources limit their grid-scale use.^3^ Aqueous zinc-ion batteries (ZIBs) offer a safer, low-cost alternative due to zinc’s abundance, high volumetric capacity (5,855 mAh cm^–3^ vs 2,062 mAh cm^–3^ for lithium), and favorable redox potential (−0.763 V vs SHE).^4^ Nonflammable electrolytes with high ionic conductivity (e.g., 50 mS cm^–1^ for 2 M ZnSO_4_) further underscore their promise for large-scale deployment.^5,6^ Various cathode materials have been explored for ZIBs, including Mn-, V-, and Co-based oxides,^7–9^ Prussian blue analogues,^10^ and layered sulfides.^11^ Organic cathodes have also become attractive due to their high capacity, tunable properties, abundance, sustainability, and chemical diversity.^12^ Examples include conjugated carbonyl compounds^13,14^ and organic conductive polymers.^15,16^

Despite advances in materials, achieving long-term stability under practical cycling conditions remains a significant challenge. Most studies reported long cycle life at high C-rate, with polymer-strengthened MnO_2_ and K-birnessite on mesoporous carbon achieving 5,000 and 6,000 cycles at 6.5C and ∼7C, respectively,^17,18^ and a polyaniline (PANI) cathode with zinc triflate electrolytes retained 91.1% capacity after 10,000 cycles at 20C.^19^ However, high-rate, low-loading studies have limited relevance for stationary storage, which requires long-term stability at low C-rates (0.2–1C) and high areal loadings.^20,21^ Under such conditions, performance is hindered by cathode dissolution, dendritic growth, and hydrogen evolution reaction (HER),^22–24^ which intensifies over extended cycling, causing gas accumulation, pH shifts, and basic zinc salt (BZS) precipitation (e.g., Zn_4_SO_4_(OH)_6_·*x*H_2_O).^24–27^ Nonuniform zinc deposition further exacerbates dendrite formation, short circuits, HER, and BZS formation, and cathode dissolution.^28,29^ Consequently, the low C-rate cycling study remains limited with ZnV_2_O_5_.nH_2_O reaching a maximum of 1,600 cycles at 1C.^30^ Using total energy throughput (TET), the product of volumetric energy density and cycle number, it is evident that many high-rate systems, despite having long cycles, deliver modest cumulative energy. For instance, a PANI/carbon cloth cathode achieves a TET of 21.4 kWh L^–1^ after 10,000 cycles, whereas our PANI/CNT cathode delivers 116.1 kWh L^–1^ after 5,928 cycles only. Sluggish ion transport, passive layer formation, electrode compaction, and mechanical stress in thick electrodes constrain high TET at low C-rates.^31,32^ Furthermore, self-discharge, a critical parameter for stationary applications, is frequently overlooked.

In this work, we address these practical aspects of stationary energy storage, moving beyond the high C-rate cycling trend. We integrate PANI with a 3D CNT sponge scaffold, leveraging the tunable redox properties, high conductivity, and reversible ion storage capability of PANI, key for efficient charge transfer in ZIBs at low C-rates. Our in-house-fabricated 3D CNT sponge,^33^ which was previously shown by our group to enhance uniform metal deposition and achieve high areal capacity for LIB anodes,^34–36^ was recently adapted for PANI cathodes in iron-ion batteries, where it improved ion transport, enhanced mechanical stability, and suppressed cathode dissolution.^37^ Additionally, modifying the zinc solvation sheath with dimethyl sulfoxide (DMSO) suppresses HER and promotes uniform zinc plating/stripping. To enhance scalability, a solvent-free dry electrode fabrication approach was applied using polytetrafluoroethylene binder. Together, these design strategies enable high-performance ZIBs that are promising for grid-scale applications.

Our ZIB comprises a PANI-incorporated CNT cathode, a commercial zinc foil anode, and 2 M ZnSO_4_ electrolyte with DMSO as an additive (Figure 1a). In the aqueous solution, each Zn^2+^ is solvated by six water molecules, forming [Zn­(H_2_O)_6_]^2+^, which leads to HER due to water instability at zinc deposition potential (Figure 1b). Additionally, ZnSO_4_ electrolyte promotes zinc dendrite growth, which can cause short circuits and further intensifies HER by exposing fresh zinc surfaces and creating localized high electric fields, thus compromising battery stability.^38^ Suppressing HER requires either altering water reduction kinetics or modifying Zn^2+^ solvation structure. DMSO, through its strong polarity and hydrogen bonding, partially replaces water molecules in the solvation shell, and raises the zinc deposition overpotential, thereby suppressing dendrite formation and mitigating HER.^39,40^ Other additives, such as glycerol and ethylene glycol (EG), similarly improve Zn reversibility via hydrogen bonding and Zn^2+^ coordination, but DMSO provides superior long-term stability at moderate current and low concentration. For instance, for 2,668 h of stable zinc plating and stripping, 68 vol % EG is required, whereas DMSO shows comparable performance at 20 vol %.^40–42^

**Figure 1:**
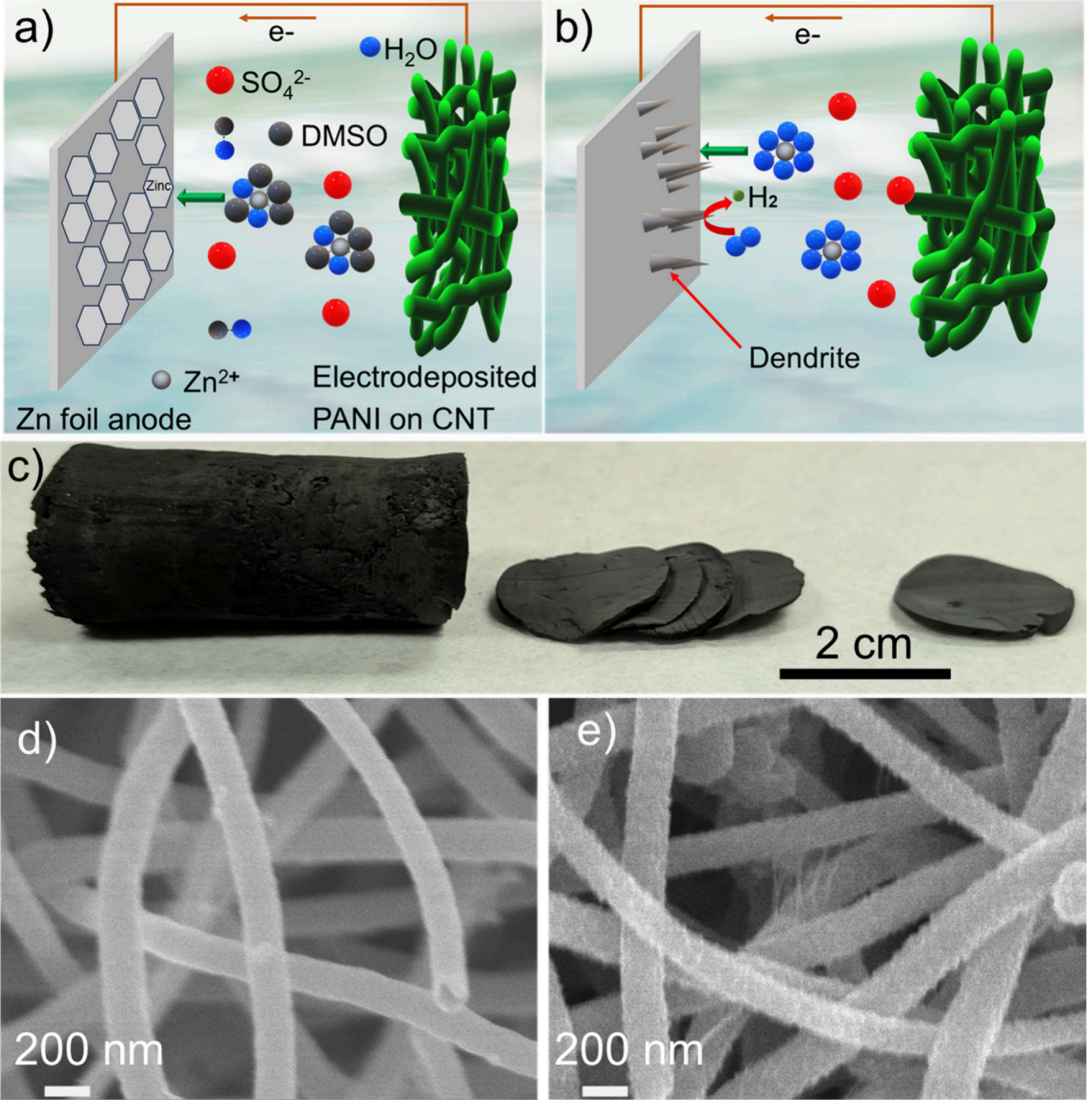
(a) Schematics of the uniform zinc deposition on the zinc anode during charging and the altered solvation structure after adding the DMSO electrolyte additive and (b) dendrite formation and hydrogen evolution on the zinc anode without the DMSO additive. (c) An as-synthesized cylindrical, sponge-like 3D interconnected CNT scaffold was sliced to electrodeposit PANI onto the fibers. SEM images of CNTs in the scaffold (d) before and (e) after electrodepositing PANI, showing changes in the diameter and morphology of the pristine CNTs.

The cathode was fabricated by electrodepositing PANI onto a sponge-like porous CNT structure (Figure 1c). Scanning electron microscopy (SEM) images show that the pristine CNTs have smooth surfaces with diameters of 150–200 nm (Figure 1d). After PANI deposition, the CNT surfaces become rougher, with diameters increasing by tens of nanometers, indicating uniform PANI coating (Figure 1e). This nanoscale surface texturing increases the surface area, thereby enhancing Zn-ion reaction kinetics. Initially, PANI formed in HCl contains partially oxidized doped (=NH^+^−) and undoped (=N−) nitrogen. During the first discharge, (=NH^+^−) in PANI is reduced to (−NH−), releasing Cl^–^, while (=N−) is reduced to (−N^–^−), interacting with Zn^2+^ ions. Upon charging, (−N^–^−), PANI oxidizes back to (=N−), releasing Zn^2+^, and (−NH−) oxidizes back to (=NH^+^−), likely interacting with (SO4)^2–^ via H-bonding instead of Cl^–^.^15^

We evaluated the impact of C-rate and PANI loading on the discharge capacities and long-term cycling (Figure 2). Figure 2a shows the effect of PANI loading (15, 22, 26, and 32 wt %) on the specific discharge capacity at 0.68C (based on the theoretical PANI capacity of 294 mAh g^–1^,^43^ with additional loadings (27.4, 47, 49, and 51.3 wt %) shown in Figures S1–S4. A loading of 15 wt % resulted in a high specific capacity of 189 mAh g^–1^. As the loading increases, the specific capacity drops. This trend is consistent with the literature, where higher capacities are often reported at low loadings.^44,45^ Lower PANI loadings preserve the CNT network for efficient transport, while higher loadings form thicker coatings that hinder electrolyte access and reduce capacity. At lower C-rates, higher initial capacities were observed, reaching ∼132 mAh g^–1^ at 0.34C and ∼158 mAh g^–1^ at 0.17C even with a much higher 51.3 wt % loading (Figure S5). The capacity went up to ∼180 mAh g^–1^ at 0.17C. Across all loadings and C-rates, capacity first rises due to gradual electrochemical activation of PANI, then stabilizes before declining. This fading is typical for PANI, arising from structural degradation, ion trapping, and mechanical stress during cycling, which progressively limit reversibility.^46–48^

**Figure 2:**
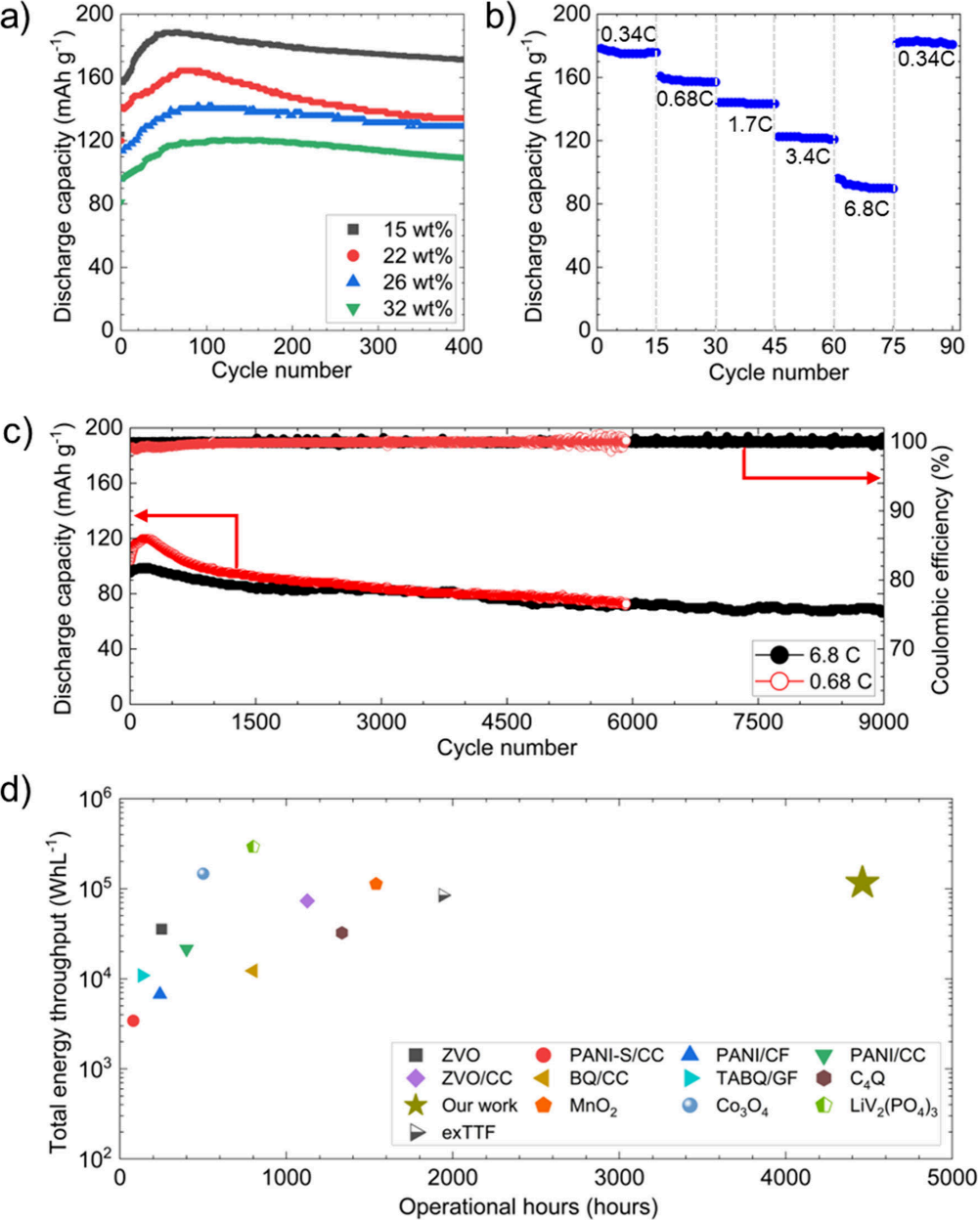
(a) Effect of PANI loading on the specific discharge capacity at 0.68C. (b) Rate capability of a cell with PANI loading of 15 wt %. (c) Long cycle life at 0.68C and 6.8C with PANI loading of 32 wt % (2 mg cm^–2^). (d) Comparison of total energy throughput for this work and the literature. See also Table S1.

The 98% porous CNT sponge accommodates high PANI loading (up to 72 wt %) without pore blockage, as SEM (Figure S5) confirms pores remain open even at 6 mg cm^–2^. This supports high active loading while preserving the CNT network’s conductivity, unlike binder-dependent slurry electrodes. Cells with PANI loadings of 3–6 mg cm^–2^ demonstrate ∼1000 cycle life with high capacity retention at low C-rates (Figures S1–S4). The rate capability test shows a typical decrease in capacity with increasing C-rate. Specifically, the cell with 15 wt % PANI loading delivered approximately 180 mAh g^–1^ at a 0.1 A g^–1^ (0.34C), 122 mAh g^–1^ at 3.4C, and 85 mAh g^–1^ at 6.8C. The initial capacity at 0.34C was fully recovered after the rate capability test. Figure 2c shows the extended cycling stability at 0.2 A g^–1^ (0.68C) and 2 A g^–1^ (6.8C) with ∼32% PANI loading (2 mg cm^–2^). Cells were cycled to 70% capacity, with the 6.8C cell lasting 8,986 cycles and the 0.68C cell 5,928 cycles, demonstrating exceptional longevity across rates. Both showed an initial capacity rise, more pronounced at 0.68C due to gradual PANI activation and interface stabilization, whereas at 6.8C the process was kinetically limited. The 0.68C cell began with lower CE that rose to ∼100% as interfaces stabilized and side reactions diminished, while the 6.8C cell showed consistently high CE, likely because faster cycling limited time at low potentials where side reactions occur.

To enable a holistic comparison of long-term performance, we benchmarked our cell’s TET against literature values, where TET is defined as

$$\mathrm{TET}=E_{\mathrm{vol}}\times N_{\mathrm{cycles}}$$

where *E*_vol_ (mWh L^–1^) is the volumetric energy density and *N*_cycles_ is the number of cycles. The volumetric energy density is given by

$$E_{\mathrm{vol}}=\frac{C_{\mathrm{sp}}m_{\mathrm{loading}}V_{\mathrm{avg}}}{t}$$

with *C*_sp_, the specific capacity (mAh g^–1^), *m*_loading_, the active material loading (mg cm^–2^), *V*_avg_, the average discharge voltage, and *t*, the combined thickness (cm) of the current collector, separator, and electrodes. TET in Wh L^–1^ is plotted against the hours of operation (actual or calculated theoretical time from the applied current density). As illustrated in Figure 2d, our work achieves nearly 5,000 h of operation with high energy throughput maintaining longevity at practical current density and outperforming prior studies that compromise runtime for high C-rate cycling (>10–20C). Notably, our comparison is based on theoretical operational hours from literature values, while our experimental data employ actual cycling times, further highlighting the superiority of our PANI/CNT cathode.

The SEM images presented in Figure 3 provide the visual evidence of the influence of DMSO as an electrolyte additive on the zinc anode morphology during prolonged cycling. As shown in Figure S6a, the initial morphology of the bare zinc anode serves as a baseline for comparison. In the absence of DMSO, Figure 3a,b clearly illustrate the detrimental effects of extended cycling, even after just 1,146 cycles. Severe dendrite formation is evident, characterized by vertical dendrite growth (Figure 3a) and, critically, separator penetration (Figure 3b). In contrast, the addition of DMSO to the electrolyte results in a significant improvement in zinc deposition. Figure 3c shows that after a comparable number of cycles (1,150 cycles), zinc deposits uniformly along the in-plane direction, demonstrating the efficacy of DMSO in suppressing dendrite formation. This observation aligns with previous reports showing that DMSO modifies the zinc solvation sheath by replacing water molecules, increasing the nucleation overpotential, and reducing the density of nucleation sites, which promotes more uniform zinc deposition,^39,49^ preferentially growing along the in-plane (101) direction, leading to a compact, layered morphology, in contrast to the vertical (002) growth.^50^ Furthermore, Figure 3d and Figure S6b demonstrate the stability of the zinc anode with DMSO after extended cycling (6,000 and 10,000 cycles, respectively). The absence of dendritic protrusions and separator attachment, highlights the effectiveness of the DMSO additive in promoting reversible zinc deposition.

**Figure 3:**
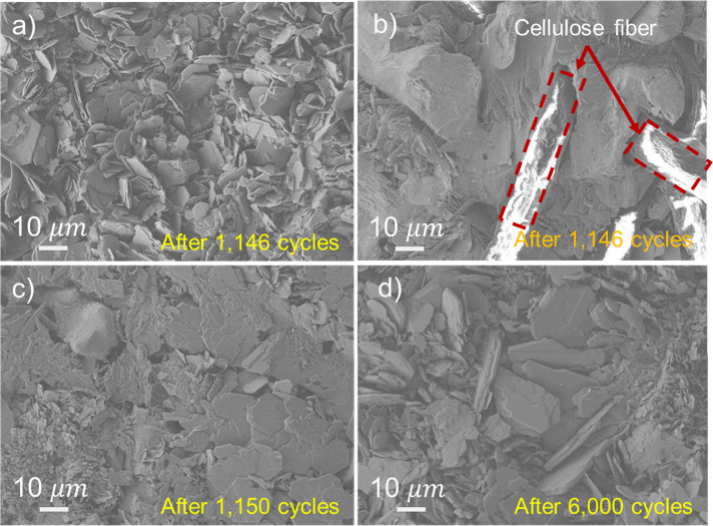
SEM images of the zinc anode after cycling under discharge conditions (a, b) without and (c, d) with 20 wt % DMSO in the 2 M ZnSO_4_ electrolytes. (a) Vertical dendrite growth toward the separator after 1,146 cycles. (b) Cellulose fibers are embedded in the dendrites, indicating dendrite penetration into the separator. With the addition of DMSO, planar zinc deposition after (c) 1,150 and (d) 6,000 cycles.

Despite the significant positive impact of DMSO on the zinc anode, it did not affect the cathode capacity, as shown in Figure S7. The cell with the DMSO additive exhibits almost identical capacity with improved stability. Previously, only 150 cycles were reported for a carbon felt/PANI cathode in a 3 M ZnSO_4_ electrolyte, due to the electrophilic nature of PANI.^15^ To stabilize the oxidized PANI, they proposed using bulky CF_3_SO_3_^–^ anions with Zn­(CF_3_SO_3_)_2_ electrolytes. However, our cell demonstrates almost 1,200 cycles at 3.4C with a lower electrolyte concentration, even without the additive. This enhanced stability can be attributed to the interconnected 3D CNT, which provides a superior conductive matrix which effectively mitigates the capacity fading commonly observed due to the high resistivity of the pernigraniline state of PANI, formed in aqueous electrolytes that results in low cycle life.^51–53^ Similar benefits of conductive matrices using reduced graphene oxide/PANI composite electrode were reported earlier.^54^ This result highlights that the use of a 3D CNT negates the need for expensive electrolytes and shows that the DMSO additive only improves the Anode. Furthermore, XRD analysis of the CNT/PANI cathode (Figure S8) revealed no evidence of BZS formation. The detection of only graphitic carbon and Zn peaks confirms that Zn^2+^ storage is confined to the cathode and that DMSO effectively suppresses these detrimental side reactions. This suppression, combined with uniform zinc plating and the CNT host’s superior properties, contributes to the observed extended cycle life.

The electrochemical performance of the assembled cell was further investigated using a combination of techniques. Cyclic voltammetry (CV) at scan rates of 0.1, 0.2, 0.5, and 1 mV (Figure 4a) reveals well-defined and reversible redox peaks, with anodic peaks observed around 1.2–1.3 V and cathodic peaks around 1.02–1.1 V. This indicates the favorable electrochemical kinetics of the cell. Analysis of the peak currents as a function of scan rate (Figure 4b) using a log­(*i*) vs log­(*v*) plot yielded *b*-values of 0.71 for the anodic process and 0.95 for the cathodic process. These *b*-values suggest that the charge storage mechanism involves a combination of diffusion-controlled and capacitive processes, with the cathodic process exhibiting a greater contribution from surface-controlled reactions. This is highly favorable for rate capability. In contrast, the anodic peak, with a *b*-value of 0.71, suggests a mixed mechanism, enhanced by the 3D CNT’s improved transport.

**Figure 4:**
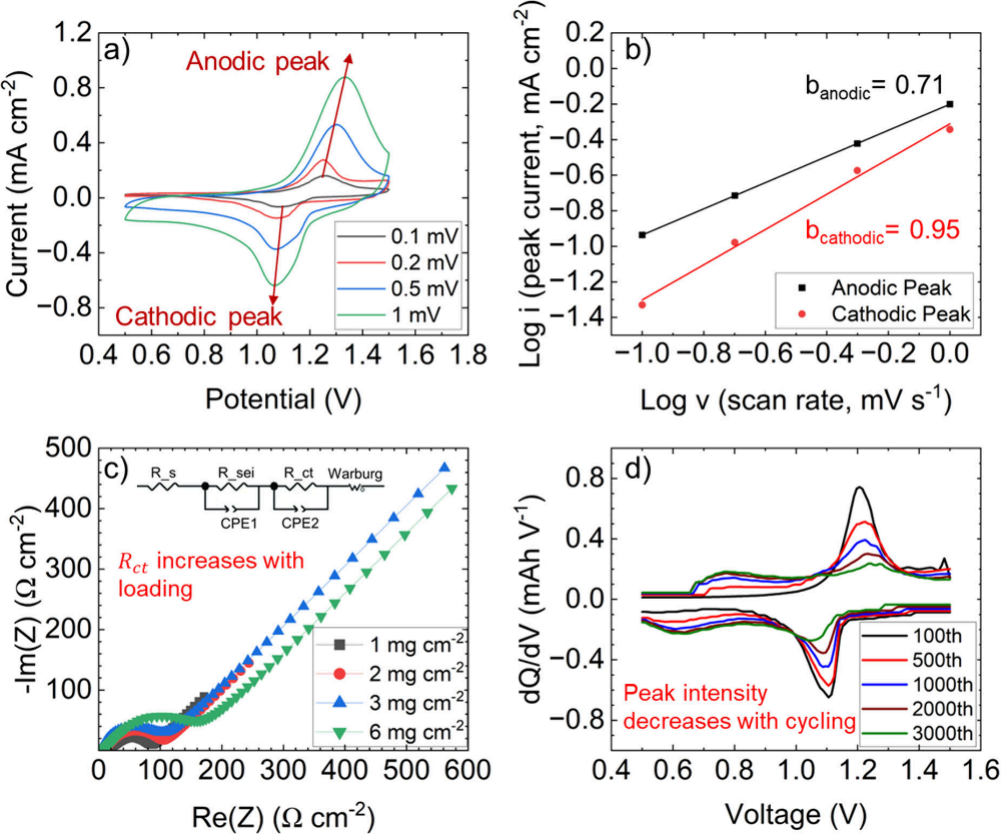
Electrochemical performance of Zn/PANI-CNT electrodes in 2 M ZnSO_4_ with 20 wt % DMSO. (a) CV curves at 0.1–1.0 mV s^–1^ show well-defined redox peaks, indicating reversible Zn^2+^ insertion/extraction. (b) log­(*i*)–log­(*v*) plots yield *b*-values of 0.71 (anodic) and 0.95 (cathodic), suggesting mixed kinetic behavior with capacitive dominance on reduction. (c) Nyquist plots (10^6^ to 0.01 Hz) for varying loadings (1–6 mg cm^–2^) show increasing impedance with mass loading. (d) d*Q*/d*V* profiles over 3,000 cycles reveal stable redox activity and almost no peak shift, confirming long-term cycling stability.

Electrochemical impedance spectroscopy (EIS) was performed to analyze the cell’s resistance characteristics. Figure 4c shows the Nyquist plots for different cathode loadings (1, 2, 3, and 6 mg cm^–2^). The high-frequency intercept with the real axis indicates a solution resistance (*R*_s_) of approximately 5 Ω cm^–2^, which remains relatively constant across different loadings. A distinct semicircle corresponding to the charge transfer resistance (*R*_ct_) was observed, while a clear signature of the solid electrolyte interphase (SEI) resistance (*R*_sei_) was either absent or too small to be resolved, potentially overlapping with R_ct_. The R_ct_ values increased with increasing cathode loading, from 75 Ω cm^–2^ at 1 mg cm^–2^ to 172 Ω cm^–2^ at 6 mg cm^–2^, indicating a slight increase in kinetic limitations with higher mass loading.

The long-term cycling stability and electrochemical reversibility were assessed using differential capacity (d*Q*/d*V*) analysis. Figure 4d presents the d*Q*/d*V* plots at various cycle numbers (100, 500, 1,000, 2,000, and 3,000 cycles). The d*Q*/d*V* profiles exhibit clear and reversible peaks, consistent with the redox reactions observed in the CV measurements. While the peak height decreases with increasing cycle number, indicating a gradual reduction in capacity, the peak positions remain relatively stable, confirming the structural reversibility of the electrode materials. These consistent peaks indicate that the fundamental redox mechanisms of the PANI cathode remain active and reversible. The gradual decrease in peak height suggests a gradual capacity fade, likely due to minor structural changes or electrolyte interactions. The stability of the peaks in the d*Q*/d*V* plots, combined with the high *b*-value for the cathodic peak in the CV analysis, highlights the effectiveness of the CNT sponge in maintaining the electrochemical activity of the PANI cathode for long-term cycling.

To further evaluate the cell’s suitability for practical applications, its self-discharge behavior and performance under intermittent rest periods were investigated. Figure 5a illustrates the cycling performance of over 100 cycles, incorporating rest intervals ranging from 12 to 204 h between normal charge/discharge sequences. Throughout rest-induced cycling, the cell exhibited consistent capacity recovery, maintaining a high CE of 90% during rest periods and recovering to >99% during normal cycling. This suggests that the cell’s electrochemical processes are highly reversible and robust under prolonged rest conditions, which is crucial for applications requiring intermittent operation. Figure 5b presents the voltage vs time curves at late-stage cycling, confirming the stability and reproducibility of the voltage plateaus during extended rest-induced cycling. The observed consistent voltage plateaus demonstrate the cell’s ability to maintain stable electrochemical performance under practical operating conditions. The reversibility of Zn^2+^ storage and the minimal hysteresis are further supported by the voltage vs capacity profiles shown in Figure 5c, which display selected cycles, 66th and 70th without intermediate rest, and the 68th cycle with intermediate rest. This indicates the efficient Zn^2+^ insertion/extraction and suggest minimal structural changes during cycling, contributing to the cell’s stability. Finally, a rigorous analysis of voltage relaxation profiles following controlled discharges to varying cutoff voltages (1.05–1.5 V, Figure 5d) elucidated the self-discharge mechanism. Increasing the depth of discharge resulted in a lower subsequent equilibrium potential, yet equilibrium was reached more rapidly. This initially larger thermodynamic driving force for parasitic reactions led to a transient self-discharge, which effectively diminished as the cell stabilized at the lower potential, indicating minimal long-term capacity fading during rest. To further investigate self-discharge behavior, we evaluated two protocols over extended operational durations: (i) standard self-discharge from a fully charged state followed by discharge and charge, and (ii) intentional discharge to intermediate voltages (1.05–1.4 V), followed by rest, full discharge (to 0.5 V), and charge. Both protocols incorporated rest periods ranging from 12 to 240 h in 12 h increments. As shown in Figure S9, the cell undergoing normal self-discharge (total duration ∼2,090 h) consistently retained a higher voltage after rest compared to the cell with intentional intermediate discharge before rest (total duration ∼1,380 h). These results suggest that initiating rest from a higher state of charge leads to better voltage retention over prolonged periods. The voltage profiles are shown in Figures S10 and S11. Collectively, these findings demonstrate the excellent electrochemical stability and reversibility of the Zn/PANI-CNT cell under various operating conditions, including intermittent rest periods and varied discharge depths.

**Figure 5:**
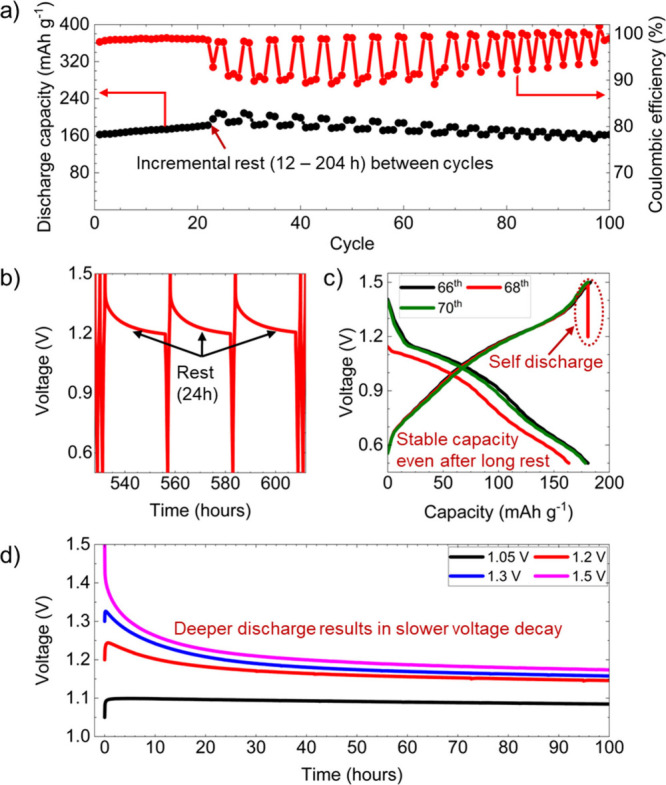
Electrochemical behavior of a Zn/PANI-CNT cell under intermittent rest periods and varied discharge conditions. (a) Cycling performance over 100 cycles with progressively extended rest intervals (12, 24, 36, ..., 204 h) inserted between charge–discharge steps demonstrate stable capacity recovery and high Coulombic efficiency throughout. (b) Voltage–time curves during rest periods display similar decay profiles across cycles, indicating consistent self-discharge behavior. (c) Voltage–capacity profiles for cycles 66 and 70 (no rest) and cycle 68 (preceded by a 24 h rest) show minimal hysteresis, highlighting reversible Zn^2+^ storage. (d) Voltage decay profiles following discharge to various cutoff voltages (1.50–1.05 V) revealing that deeper discharge results in slower decay and faster stabilization, while higher cutoff voltages lead to faster initial decay but stabilize at higher equilibrium potentials.

Beyond self-discharge behavior, practical energy storage systems must also exhibit resilience to electrical faults such as short circuits. Our beaker-type Zn/PANI cells showed full recovery after deliberate short-circuiting (internal and external), confirming nonflammability, robustness, and inherent safety (Figures S12 and S13). Even at 2 mg cm^–2^ and 6.8C, the beaker cell sustained >1,000 cycles (Figure S1), demonstrating that long cycle life translates from coin cells to scalable formats. The design allows optimization with thin separators for modular assembly and replaceable electrodes for long-term use.

To further enhance scalability, a dry electrode fabrication approach was developed using CNT chunks derived from our 3D CNT. This solvent-free approach yields mechanically robust electrodes with a capacity of ∼140 mAh g^–1^ for 300 cycles at 0.5C with near 100% CE with a capacity retention of 83% of the maximum capacity (Figure S14). The initial low capacity might be due to the hydrophobicity introduced by the polytetrafluoroethylene binder; however, upon cycling, the electrode wetting improves and capacity increases. Dry-processed large-format electrodes may face challenges in thickness uniformity, adhesion, and electrolyte wetting. Strategies such as CNT functionalization, reduced binder content, controlled porosity, and mild calendaring can enhance ionic accessibility, maintain electronic contact, and mitigate trade-offs between hydrophobicity and infiltration, enabling scalable high-loading ZIBs. Beyond electrochemical performance, the freestanding dry electrodes demonstrated excellent mechanical flexibility, retaining structural integrity when bent or suspended (Figure S15a–c). SEM and energy dispersive spectroscopy (EDS) analyses further confirmed a uniform CNT distribution and well-integrated electrode architecture, supporting efficient charge transport and structural stability (Figures S15d–e, S16). This dry electrode fabrication technique, coupled with the demonstrated safety and electrochemical performance, highlights the potential of our Zn/PANI battery for stationary energy storage applications.

In summary, we present a high-performance Zn/PANI battery enabled by a 3D CNT host for the cathode, achieving long-term cycling stability at both low (5,928 cycles at 0.68C) and high (8,986 cycles at 6.80C) current density with minimal capacity fading. The porous, mechanically resilient, and conductive CNT network ensures structural integrity and uniform electron transport, while the addition of DMSO enhances interfacial stability and suppresses dendrite formation. Furthermore, the battery exhibits low self-discharge, retains over 90% of its original capacity after extended rest, and demonstrates inherent safety under short-circuit conditions. To enhance scalability, we demonstrated a dry electrode fabrication approach, yielding electrodes with high areal loading (7.9 mg cm^–2^) and a capacity of ∼140 mAh g^–1^ at 0.5C, with nearly 100% CE. Overall, our results establish a high-performance, safe, and scalable Zn-ion battery architecture, addressing key challenges in battery longevity, safety, cost-effectiveness, and environmental impact, making this system highly promising for practical deployment in large-scale stationary energy storage applications.

## Supplementary Material



## Notes

Materials and experimental methods; supplementary electrochemical data; SEM, and XRD characterization; supporting tables; references (PDF)
