# Supplementary material for: High-Areal-Loading Zinc-Ion Batteries with Long-Term Cycling at Practical Current Densities with Scalable Electrode Design
Source: Nano Lett. 2025 Sep 29;25(40):14710–6. doi: 10.1021/acs.nanolett.5c03864 (PMC12512180; doi:10.1021/acs.nanolett.5c03864)
Supplement: Supplementary file 1 [file nl5c03864_si_001.pdf]

Supplementary Information

High-Areal-Loading Zinc-Ion Batteries with Long-Term Cycling at Practical Current Densities with Scalable Electrode Design

Md Zahidul Islam[1], Choongho Yu[1,2]*

[1] Department of Mechanical Engineering, Texas A&M University, College Station, Texas, 77843 USA

[2] Department of Materials Science and Engineering, Texas A&M University, College Station, Texas, 77843 USA

* Corresponding author: chyu@tamu.edu (CY)

Materials and Methods:

CNT Fabrication

The cylindrical CNT sponge was grown inside a 1-inch quartz tube (22 mm inner diameter) in a 3-zone tube furnace (Lindberg Blue M, Thermo Scientific) using chemical vapor deposition (CVD). A detailed description is provided in our earlier work [1, 2] . In summary, 400 mg Ferrocene (Acros) was used as a catalyst for the CNT growth and was placed in zone 1. The tube was initially purged with Ar (Airgas, 99.999%), and then zone 1 and zone 3 were ramped up to 120 °C and 650 °C within 20 and 10 minutes, respectively, while flowing 260 sccm of H_2 (Airgas, 99.999%). After that, the CNT growth reaction was carried out by introducing 80 sccm of Ar, 80 sccm of C_2H_4 (Airgas, 99.999%), and 260 sccm of H_2. Argon was supplied through a bubbler that enabled the flow of water vapor with Ar flow. Intertwined CNTs were grown from the inner wall of the quartz tube and eventually fully blocked by the highly porous (98%) cylindrical CNT within 30 minutes. After the reaction, the furnace was naturally cooled to room temperature, flowing Ae. The length of the sponge is about 10 cm.

Cathode Fabrication

The CNT sponge is then cut into 80 – 100-micron-thick slices using a blade and made into circular pieces 0.71 cm^2 and 1 cm^2 circular pieces. The circular sponge was then attached to graphite foil using carbon paint and used in a two-electrode setup for the electrodeposition of Polyaniline (PANI). Graphite foil served as the counter electrode. The deposition was performed via the oxidation of Aniline (0.1M in 1M HCl) at a constant current of 1 or 2 mA cm^{-2} [3]. The PANI-

deposited CNT was washed using deionized water and dried in a vacuum oven at 60 °C. The dried PANI-deposited CNT was then used as the cathode.

Cell Fabrication

CR2032 coin cells were assembled using zinc foil (200 microns) as the anode, PANI/CNT as the cathode, Whatman qualitative filter paper (grade 1) as the separator, and 2M $ZnSO_4$ with 20 wt.% DMSO as the electrolyte. The coin cells were cycled from 0.5V to 1.5V at 0.1 – 2 A g^{-1}.

Dry Cathode Fabrication

The dry cathode fabrication process began with high-speed ball milling (SPEX SamplePrep 8000M Mixer/Mills) of the CNT sponge for 10 minutes, followed by sieving to collect CNT chunks ranging from 150 to 206 μm. These CNT chunks were then thoroughly mixed with Polyaniline (emeraldine base, molecular weight of ~50,000, Sigma Aldrich) powder using a mortar and pestle to ensure uniform dispersion. Next, Polytetrafluoroethylene (PTFE) (PTFE preparation, 60 wt.% dispersion in H_2O, Sigma Aldrich) powder was prepared by washing the suspension multiple times with deionized water and dried on a hot plate. The collected powder was then added as a binder, and the mixture was further blended until it formed a cohesive flake. This flake was then passed through a calendaring machine to achieve the desired thickness of 100 μm, forming a flexible, freestanding electrode film. Finally, the film was cut into circular discs and used as the cathode, eliminating the need for solvent-based slurry processing and enabling a scalable, environmentally friendly electrode fabrication approach. The electrode composition consisted of 87 wt.% PANI, 10 wt.% CNT, and 3 wt.% PTFE.

Short-circuit Test

The short-circuit test was carried out by placing the CNT/PANI cathode attached to a graphite foil using carbon paint in a clear container measuring 1 cm by 5 cm by 0.8 cm. The zinc foil was placed approximately 7 mm apart from the cathode. After standard discharge/charge cycles and then a 20-second rest period, the internal short-circuit test was initiated by tilting the anode and the cathode into contact. After 20 minutes, the electrodes were separated by returning them to their original positions. For the external short-circuit test, the electrodes were connected externally using an electrical wire.

Electrochemical Measurements

The electrochemical performance of the fabricated electrodes was evaluated using a Landt battery tester (CT3002AU) for galvanostatic charge-discharge (GCD) cycling. Tests were conducted within a voltage window of 0.5 – 1.5 V at various current densities, as specified in the main text. Cyclic voltammetry (CV) measurements were performed using a CH Instruments electrochemical workstation (CHI604D, Electrochemical Analyzer) within the same voltage window (0.4 – 1.6 V) at a scan rate of 0.1, 0.2, 0.3, 0.5, and 1 mV s^{-1}. Electrochemical impedance spectroscopy (EIS) was also carried out using the same CHI604D electrochemical analyzer. The frequency range for EIS measurements was 10^6 Hz to 0.01 Hz with a sinusoidal voltage amplitude of 5 mV at the open circuit potential. All electrochemical tests were performed at room temperature (approximately 25 °C).

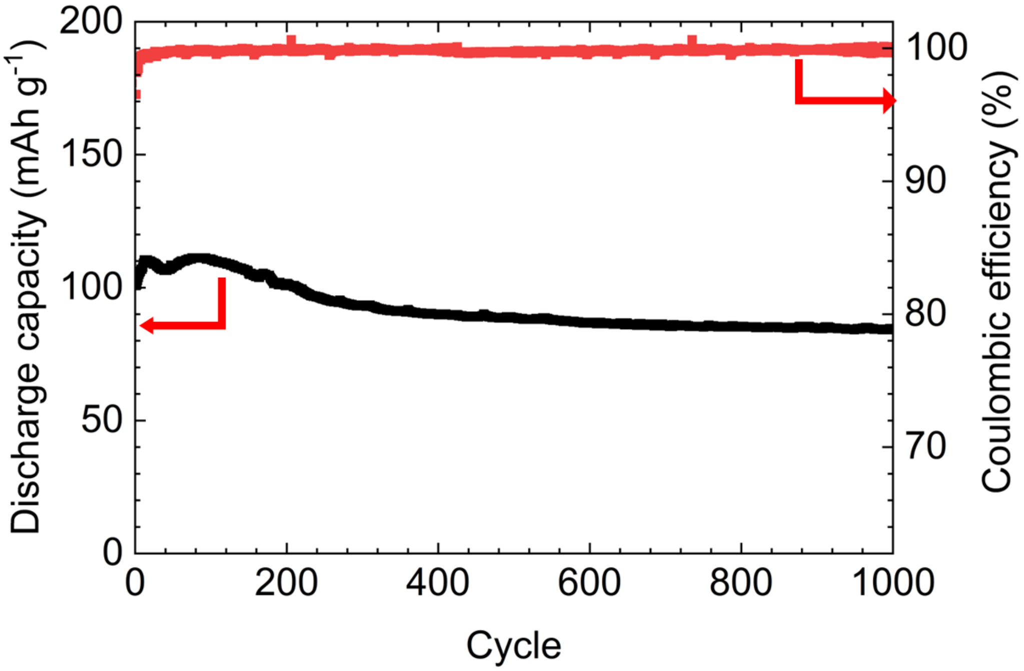

Figure S1. The cycling performance of the beaker-type cell at a 6.8C rate with an areal loading of 2 mg/cm^2 (27.4 wt.%) , with the electrodes placed approximately 1 cm apart, demonstrates robust cyclability. This indicates that the cell maintains stable performance even under high discharge rates and with a relatively wide electrode spacing, highlighting its efficiency and reliability for practical applications that require fast charging and discharging capabilities.

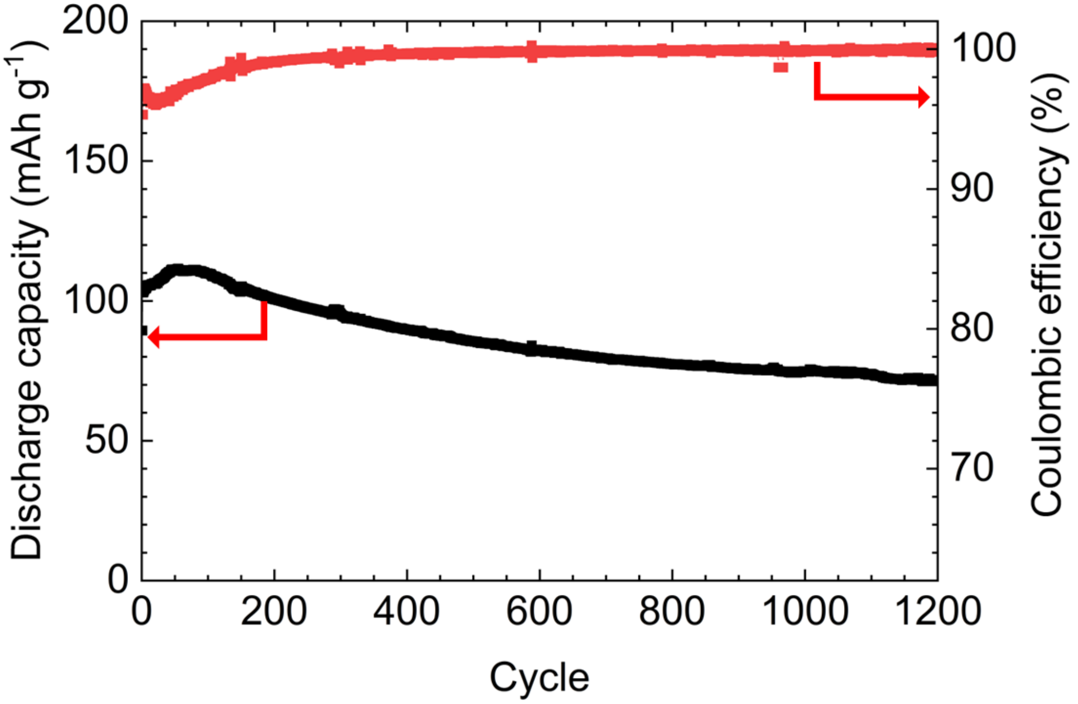

Figure S2. cycling performance of cells with a constant current density of 0.68C (47 wt.% PANI loading, 3 mg/cm^2).

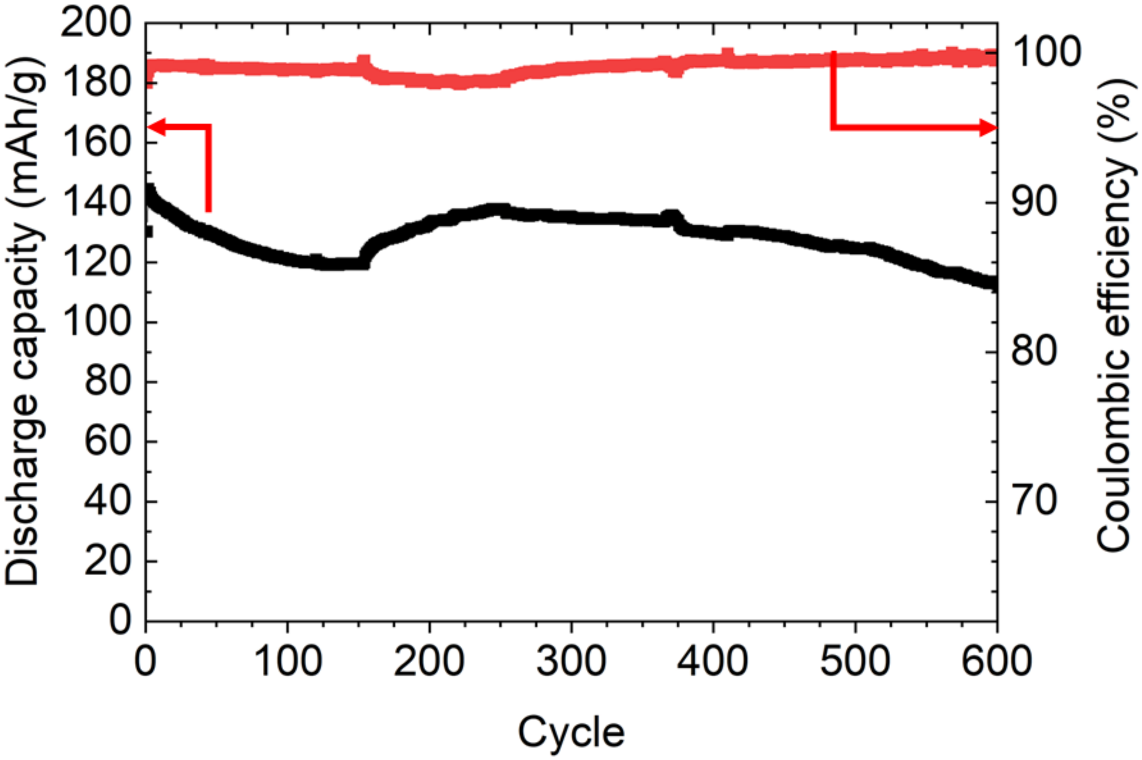

Figure S3. cycling performance of a cell with a constant current density of 0.68 C at a very high active material areal loading of 6 mg/cm^2 (49 wt.% PANI loading)

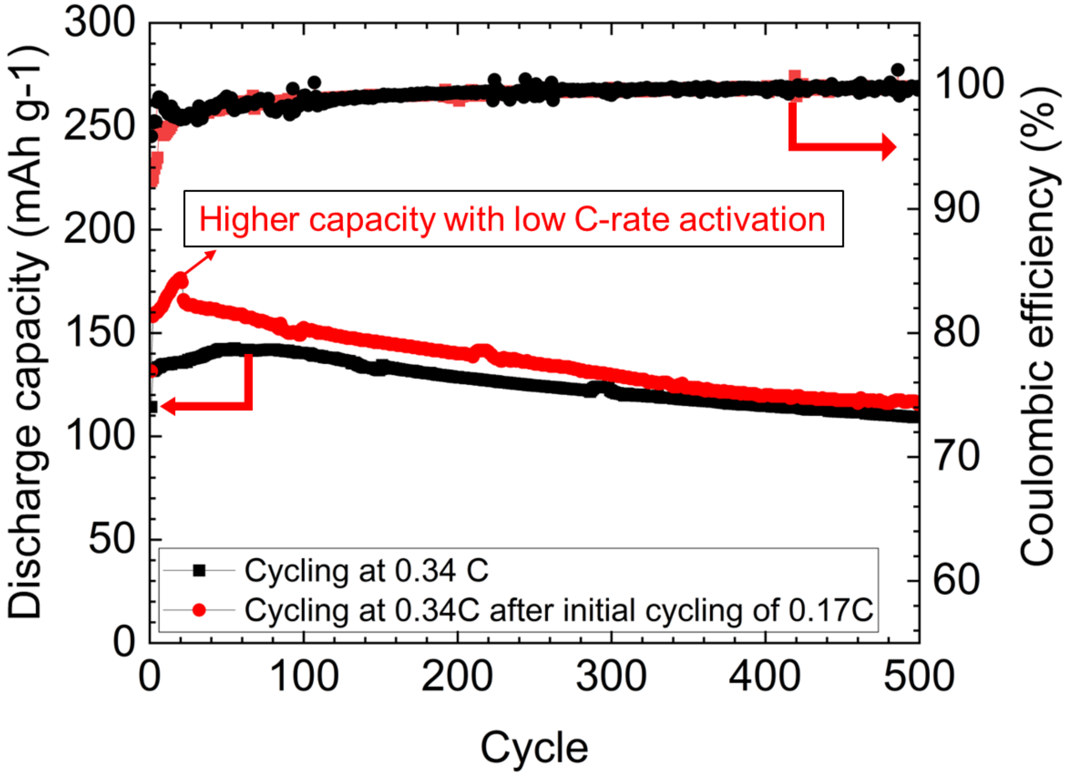

Figure S4. Capacities with the same areal loading of 3 mg cm^{-2} (51.3 wt.%) but different initial current densities (0.17 C and 0.34 C). Initial cycling with low current density shows higher than the counterpart due to better activation of the active material.

Table S1. Comparison of Total Energy Throughput (TET) with Reported Cathode for ZIB

Cathode	Active material loading (mg cm^{-2})	Volumetric Energy Density (Wh L^{-1})	Cycle Number	Total Energy Throughput (TET) (Wh L^{-1})	Time of Operation (hours)*	Ref
CNT/PANI	1.1	19.6	5928	116,118.8	4460	**Our Work**
ZVO	0.7	35.6	1000	35,557.2	250	[4]
PANI-S/CC	1.1	1.7	2000	3,420.0	80	[5]
PANI/CF	1.1	2.2	3000	6,724.5	240	[6]
PANI/CC	1.1	2.1	10000	21,423.7	400	[7]
ZVO/CC	1.4	9.8	7500	73,186.1	1126	[8]
BQ-CC	1.1	24.5	500	12,267.4	800	[9]
TABQ-GF	0.8	10.9	1000	10,886.0	133.5	[10]
C_4Q	1	32.4	1000	32,365.4	1333.5	[11]
MnO_2	1.4	22.6	5000	112,903.2	1538.5	[12]
Co_3O_4	1.6	29.3	5000	146,618.2	500	[13]
$LiV_2(PO_4)_3$	1.3	101.6	4000	406,250	800	[14]
exTTF	1	8.7	9700	84,571.9	1940	[15]

*For references without operational hours, theoretical hours were used based on cycle numbers and C-rate.

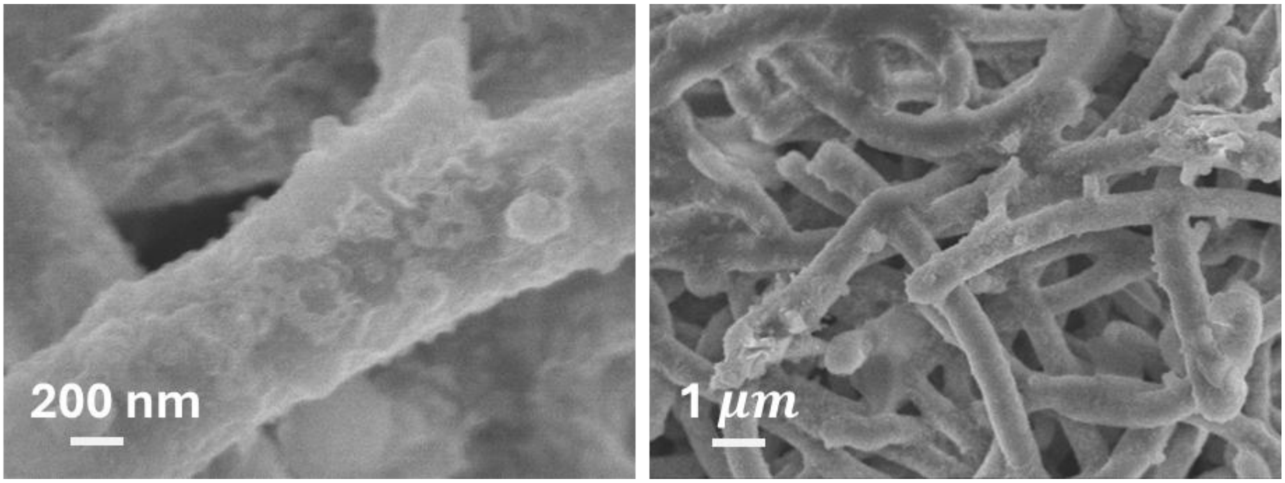

Figure S5. SEM images of the CNT/PANI cathode demonstrate the highly porous structure of the CNT even at a high areal loading of 6 mg/cm².

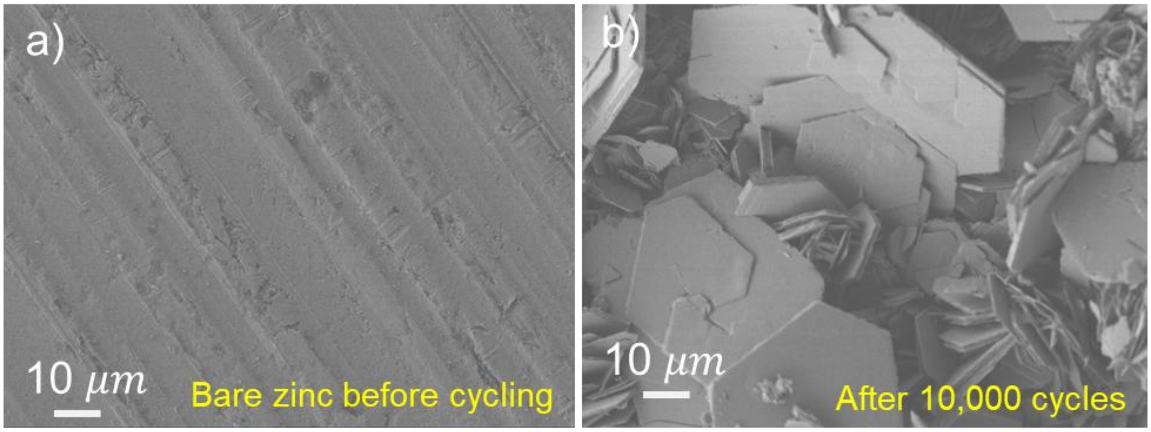

Figure S6. a) SEM image of the bare zinc, and b) a mix of planar and vertical alignment after 10,000 cycles, but without localized protrusions with DMSO additive in the electrolyte

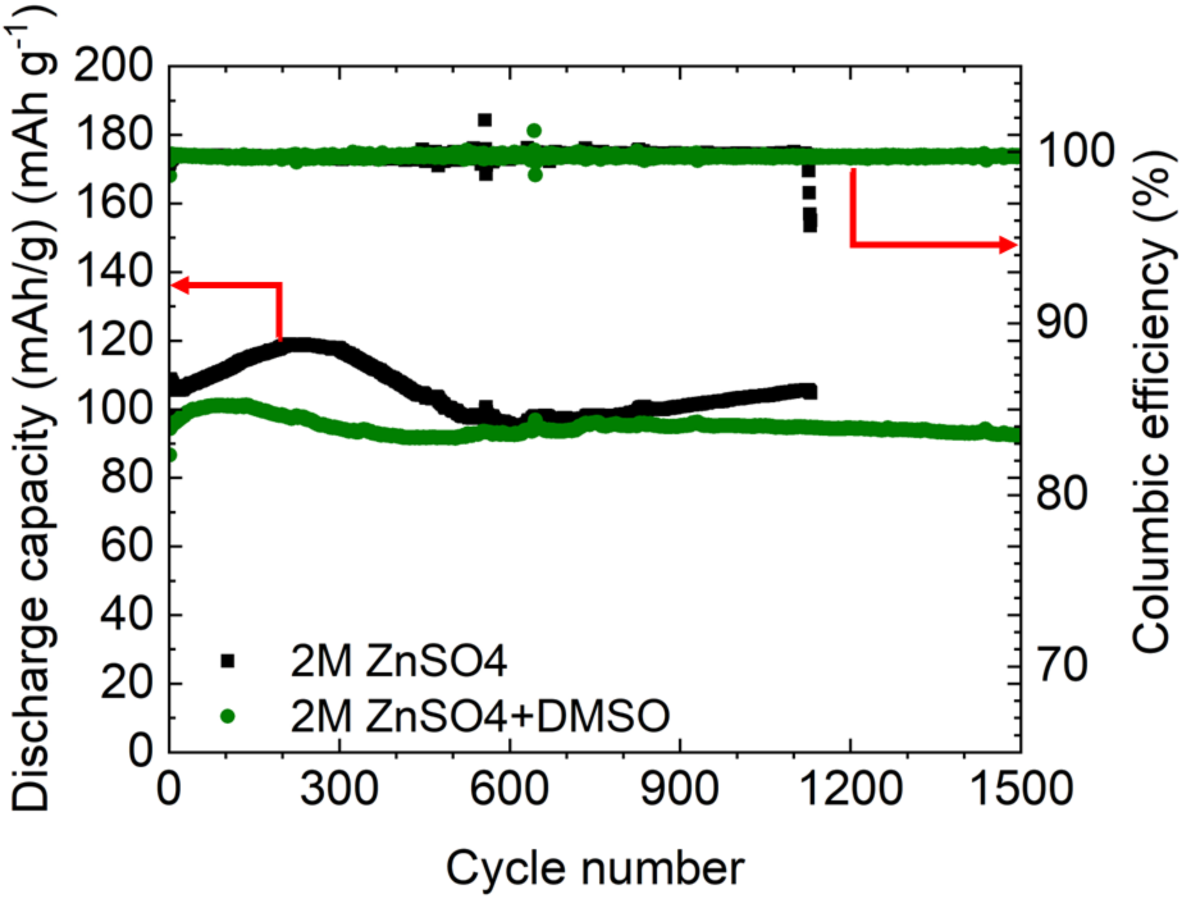

Figure S7. A comparison of cycle life and specific discharge capacity between cells with and without the DMSO additive in 2M $ZnSO_4$ electrolyte. Both the cells exhibit identical capacity at a PANI loading of 21.5 wt.% and cycled at 3.4C, however, the cell without the additive, fails due to short-circuit before reaching 1,200 cycles.

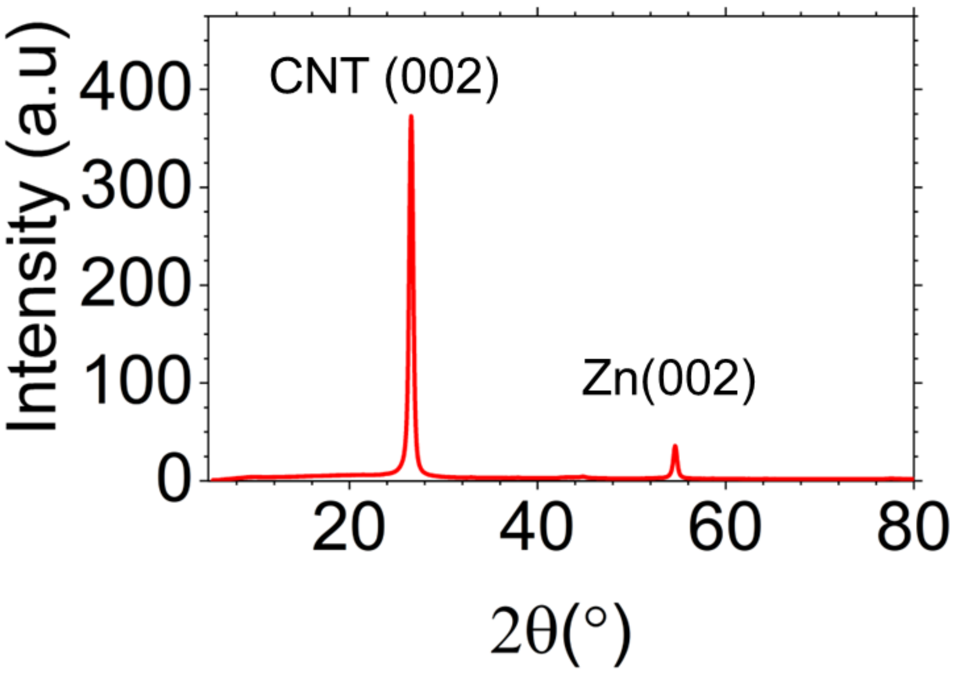

Figure S8. XRD peaks of the CNT/PANI cathode demonstrate only CNT and Zn peak demonstrating the absence of zinc hydroxyl sulfate.

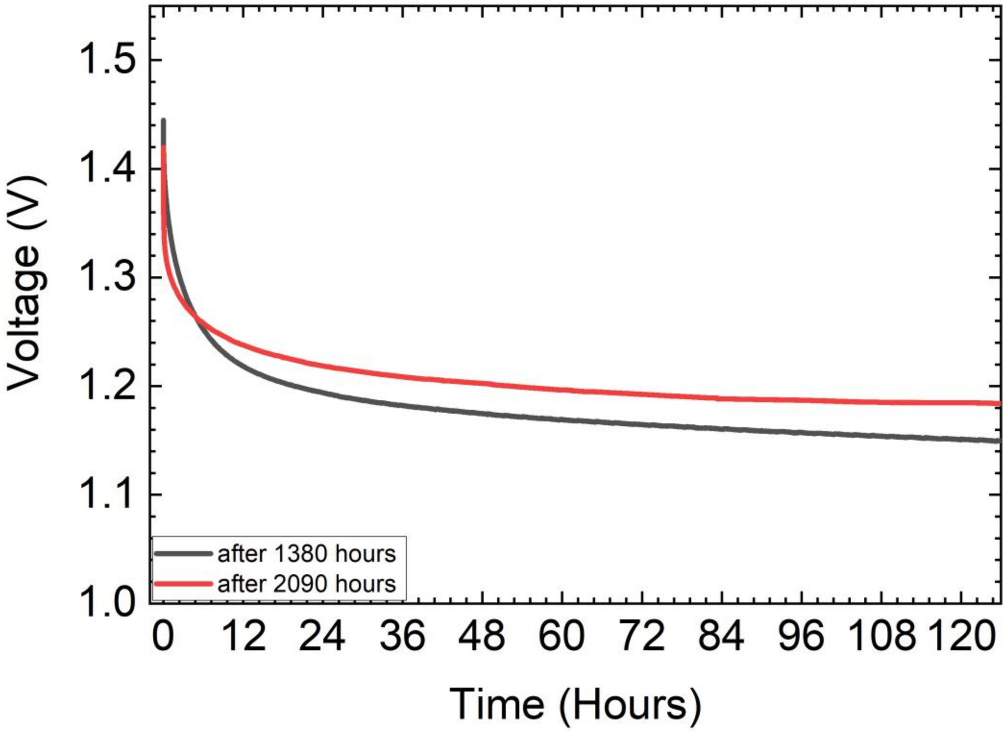

Figure S9. Voltage profiles after 1,380 hours with intentional discharge to an intermediate voltage (1.05 – 1.4 V)-rest (12 hours – 240 hours)-discharge (0.5V)-charge (1.5V) cycle and 2,090 hours of self–discharge (12 hours – 240 hours)–discharge–charge cycle. Normal self-discharge retains a higher voltage than intentional discharge to a certain voltage and rest.

.

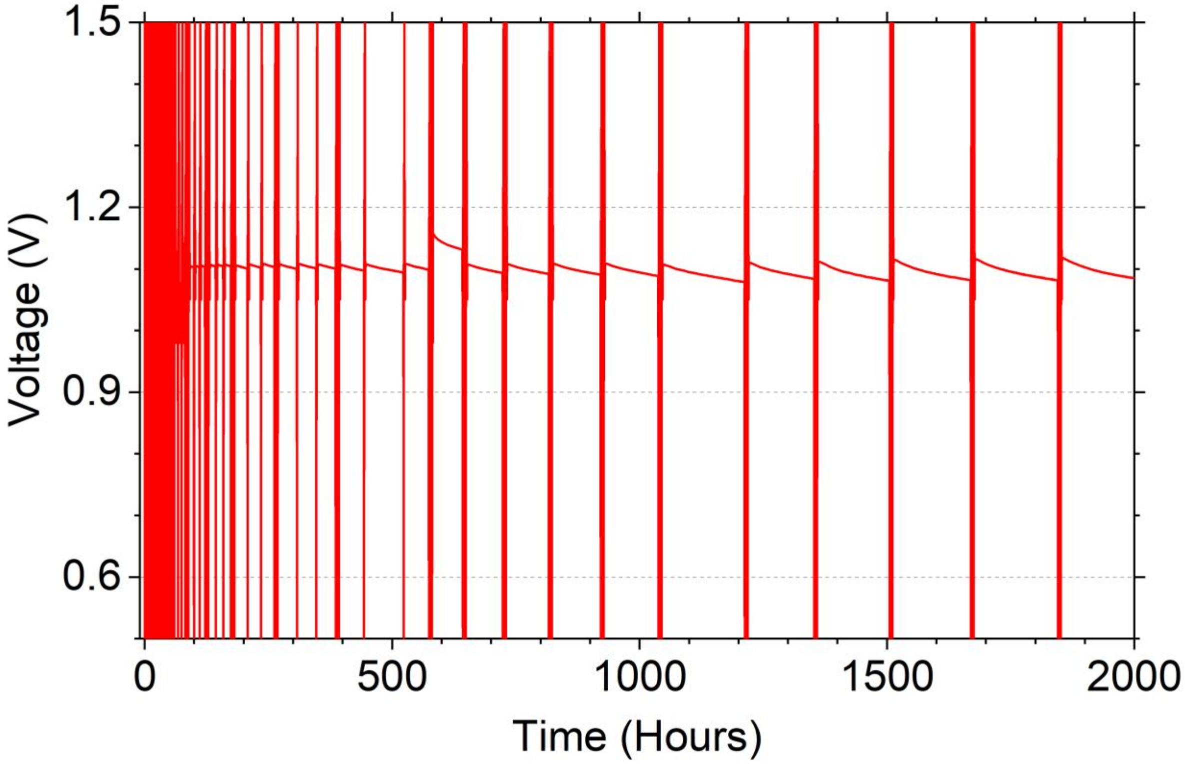

Figure S10. Self-discharge behavior of the cell with an incremental rest time (4 hours – 150 hours) after discharge to 1.05 V. The cell maintains a voltage of about ~1.1V after the rest if intentionally discharged to 1.05 V.

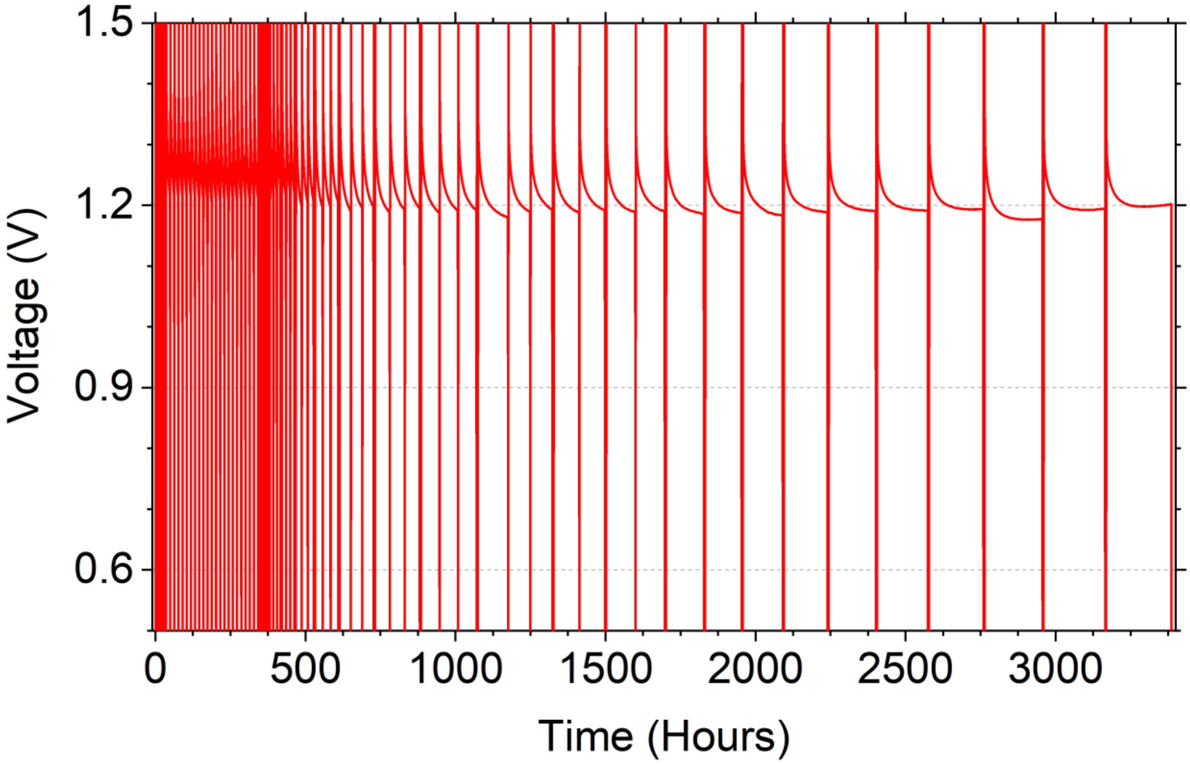

Figure S11. Self-discharge behavior of cells with an incremental rest period from 4 hours – 150 hours resting from various initial voltages (1.1 – 1.4 V). The cell maintains a voltage of about 1.2 V even after prolonged rest periods.

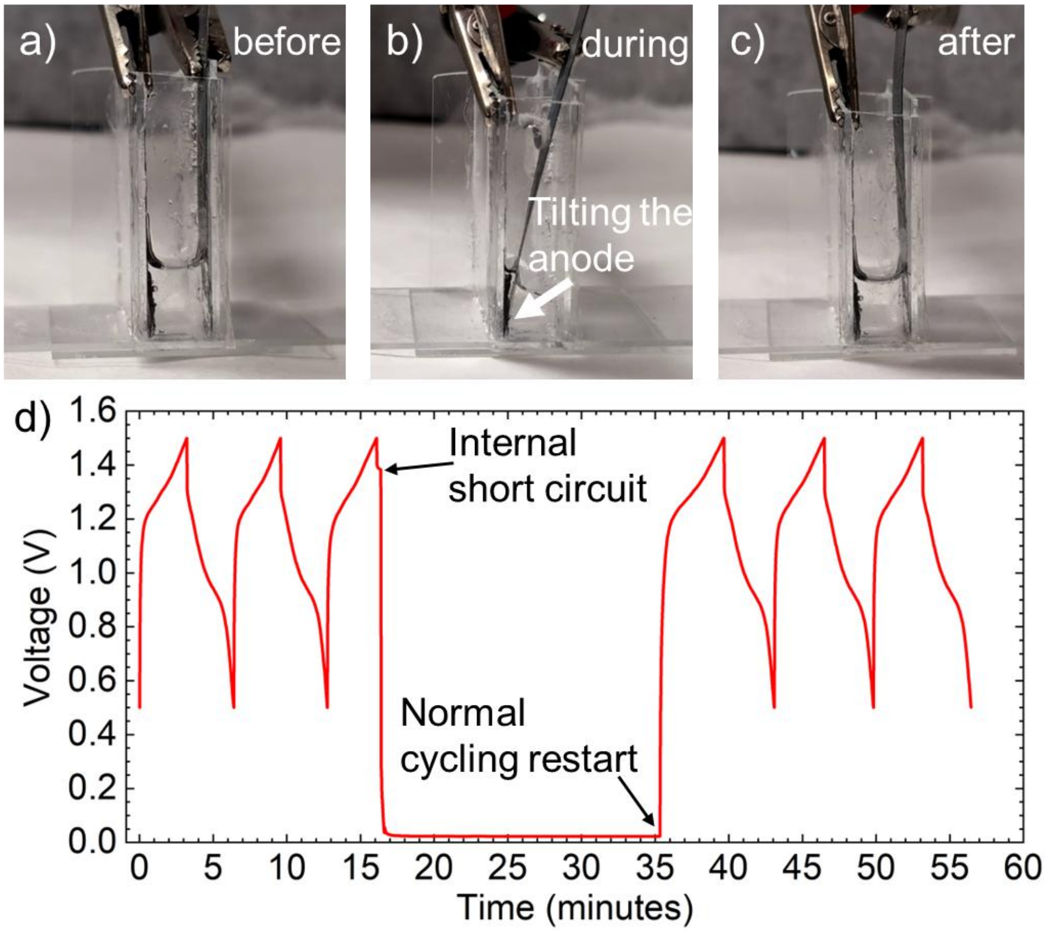

Figure 12. A beaker-type cell (a) before, (b) during, and (c) after the internal short-circuit, respectively by tilting the anode towards the cathode to make contact, and (d) the corresponding voltage profile.

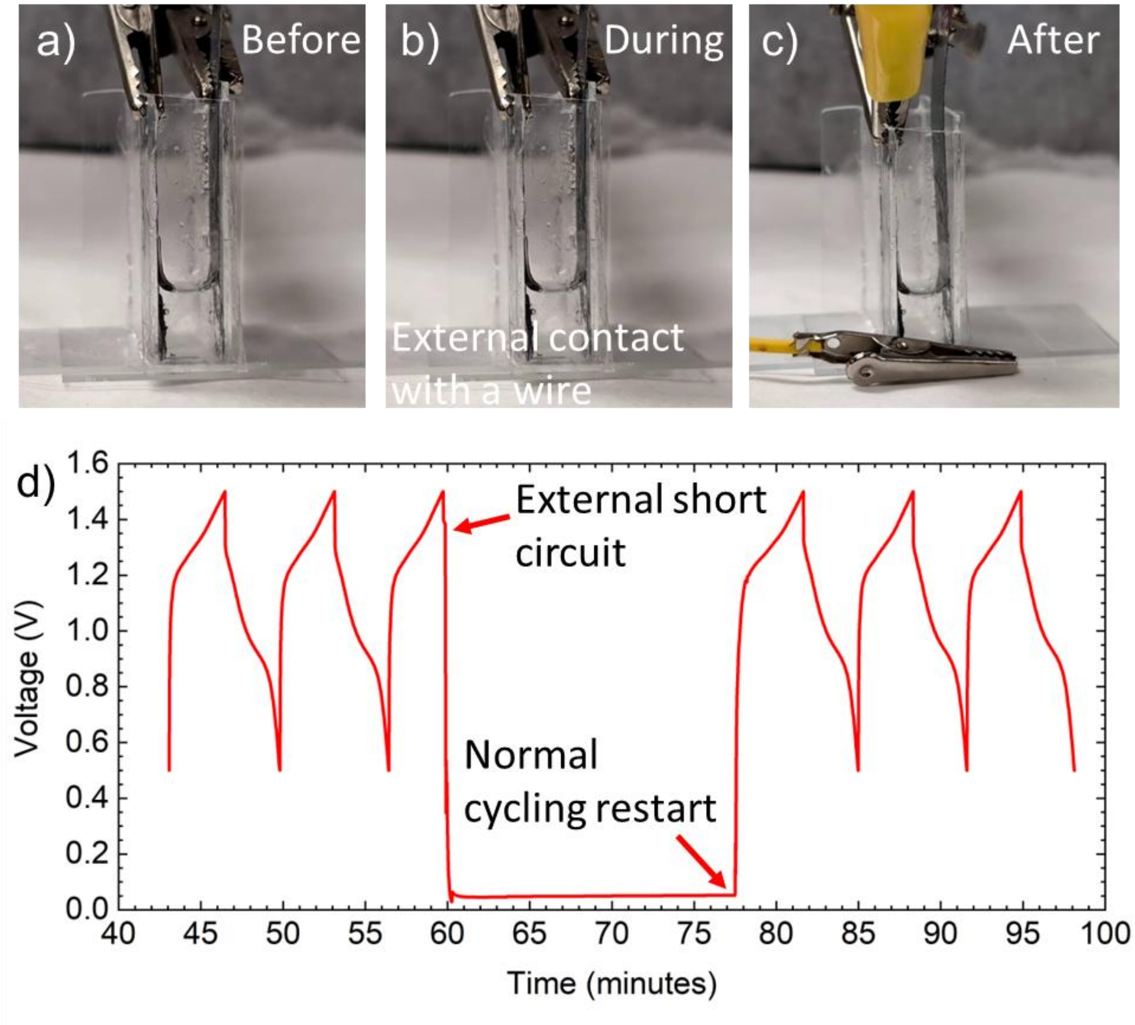

Figure S13. a), b), c) the beaker-type cell before, during, and after the external short-circuit (caused by connecting the anode and cathode with an external wire), respectively, and d) the corresponding voltage profile.

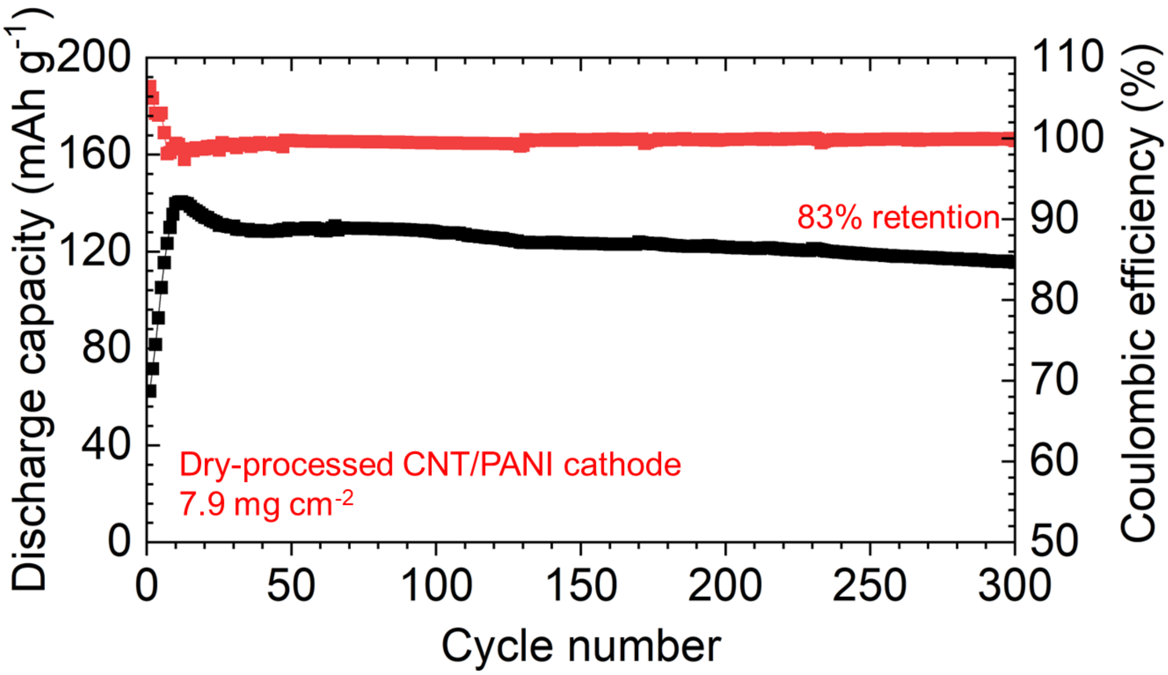

Figure S14. Cycling performance of the dry-processed CNT/PANI cathode at 0.5C for 300 cycles, demonstrating stable cycling with a capacity retention of 83% of the maximum capacity after 300 cycles.

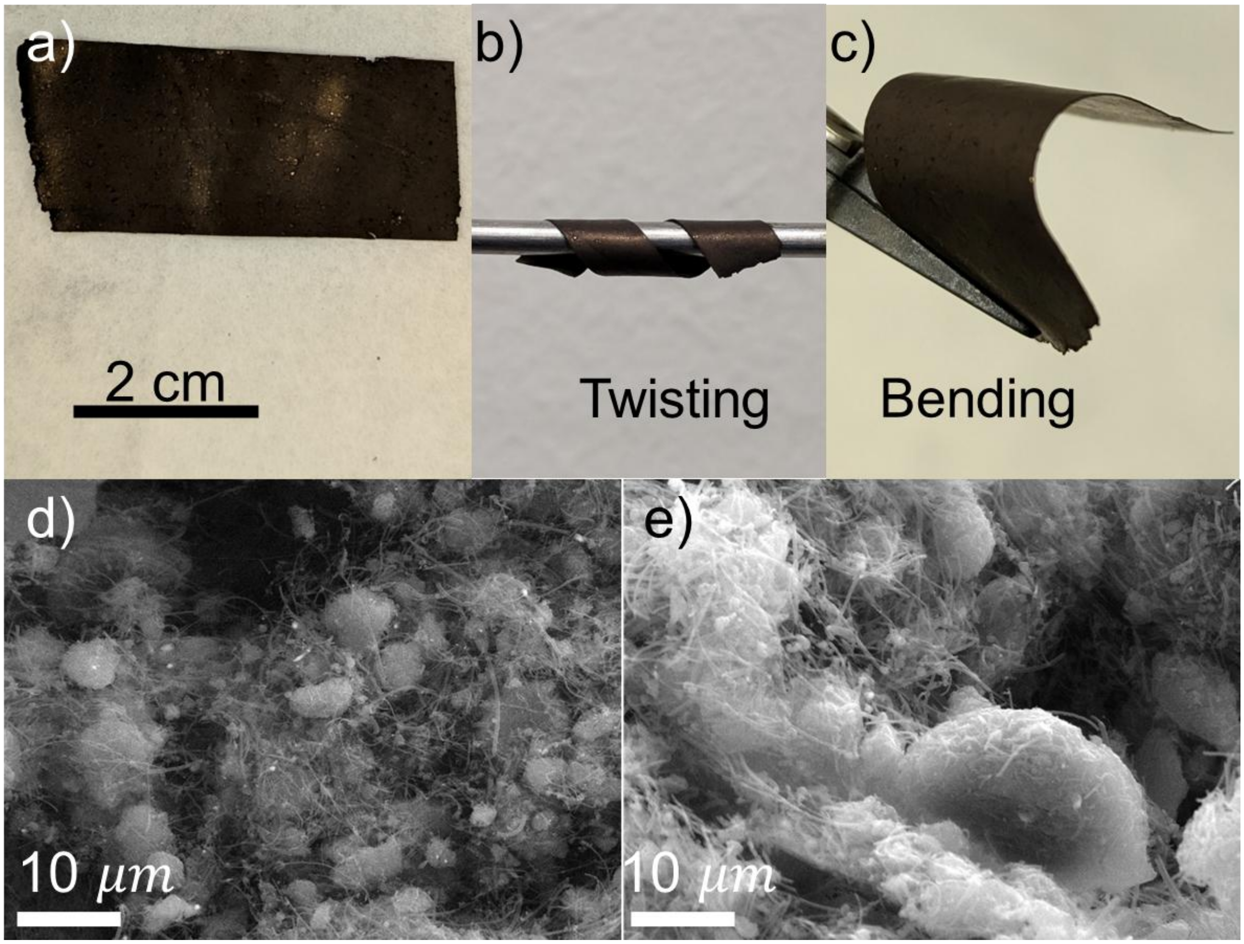

Figure S15. (a–c) Photographs of a freestanding electrode, highlighting its high flexibility and mechanical resilience by hanging from tweezers and curling around them without structural damage. SEM images of (d) the top surface and (e) cross-section, showing a uniform dispersion of CNTs and PTFE binders, which facilitates a well-connected electrical network for efficient charge transport and mechanical stability.

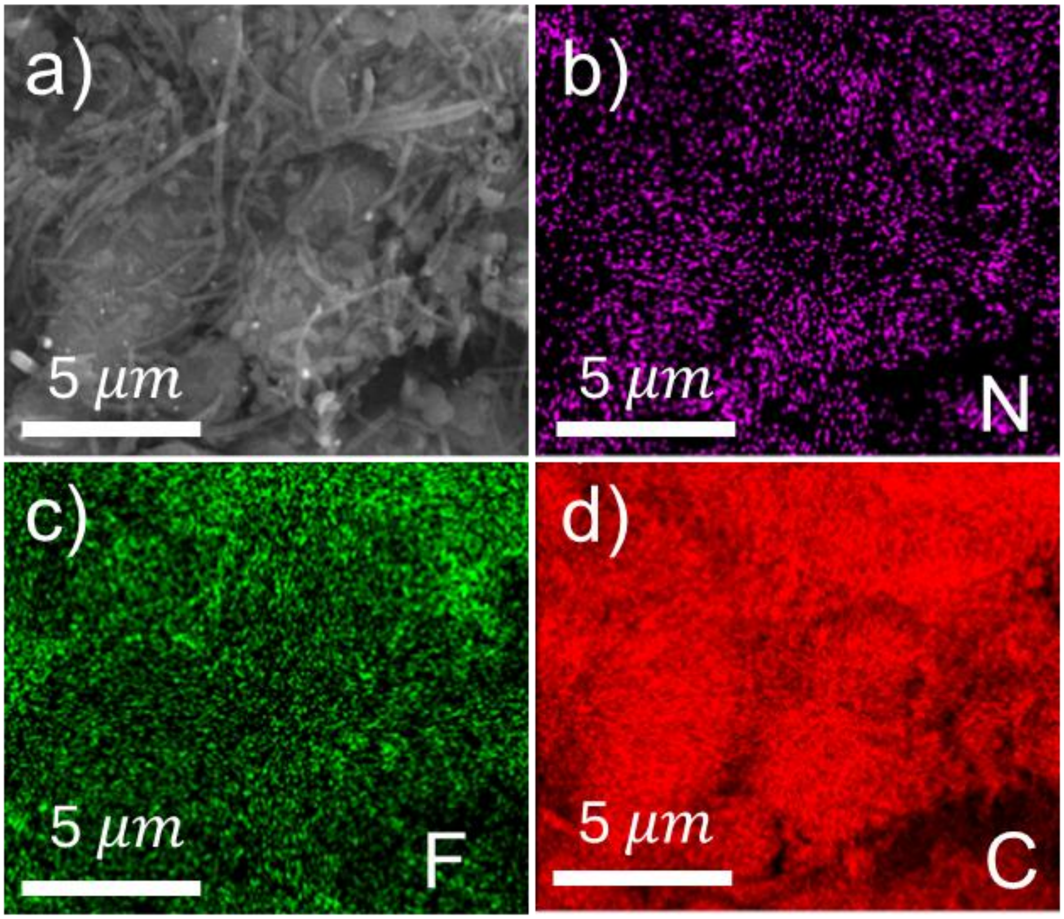

Figure S16. (a) SEM image of the dry-processed electrode showing uniformly distributed CNTs. (b–d) EDS mapping results for N (purple), F (green), and C (red) elements.

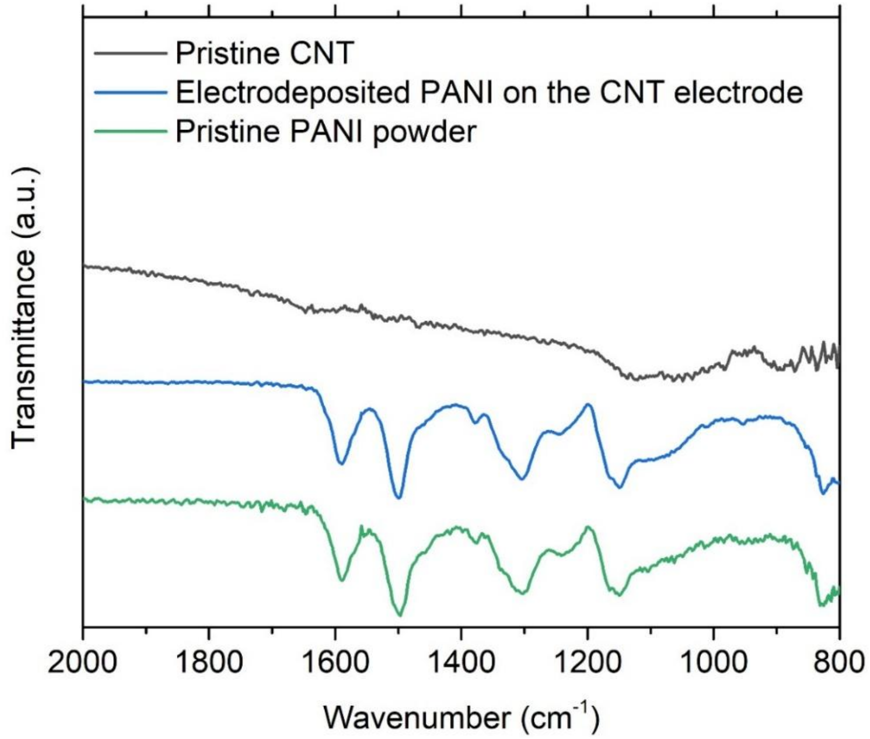

Figure S17. ATR-FTIR (Fourier transform infrared spectroscopy with attenuated total reflectance) spectra of a pristine CNT sponge, electro-deposited PANI on the CNT, and as-purchased PANI powders. Reproduced with permission from [16]. Copyright [2025] [Royal Society of Chemistry].

Table S2. Diameter, thickness, loading and areal loading of the cathode material used.

Sl. No.	Loading (wt.%)	Areal loading (mg/cm^2)	Diameter (mm)
1	15	0.56	10 mm
2	22	0.85	10 mm
3	26	1.27	10 mm
4	32	1.97	10 mm
5	28	2	10
6	49	6	10 mm
7	51.3	3	10 mm
8	47	3	10 mm
9	27.4	2	16 mm

References

1. Yang, G., et al., *Scalable synthesis of bi-functional high-performance carbon nanotube sponge catalysts and electrodes with optimum C–N–Fe coordination for oxygen reduction reaction.* Energy & Environmental Science, 2015. **8**(6): p. 1799-1807.

2. Yang, G., et al., *Bifunctional nano-sponges serving as non-precious metal catalysts and self-standing cathodes for high performance fuel cell applications.* Nano Energy, 2016. **22**: p. 607-614.

3. Kim, S.L., H.T. Lin, and C. Yu, *Thermally chargeable solid-state supercapacitor.* Advanced Energy Materials, 2016. **6**(18): p. 1600546.

4. Kundu, D., et al., *A high-capacity and long-life aqueous rechargeable zinc battery using a metal oxide intercalation cathode.* Nature Energy, 2016. **1**(10): p. 1-8.

5. Shi, H.Y., et al., *A long-cycle-life self-doped polyaniline cathode for rechargeable aqueous zinc batteries.* Angewandte Chemie, 2018. **130**(50): p. 16597-16601.

6. Wan, F., et al., *An aqueous rechargeable zinc-organic battery with hybrid mechanism.* Advanced Functional Materials, 2018. **28**(45): p. 1804975.

7. Fu, X., et al., *Polyaniline nanorod arrays as a cathode material for high-rate zinc-ion batteries.* ACS Applied Energy Materials, 2020. **3**(12): p. 12360-12367.

8. Ma, L., et al., *Achieving both high voltage and high capacity in aqueous zinc-ion battery for record high energy density.* Advanced Functional Materials, 2019. **29**(46): p. 1906142.

9. Luo, Z., et al., *High energy density aqueous zinc–benzoquinone batteries enabled by carbon cloth with multiple anchoring effects.* Journal of Materials Chemistry A, 2021. **9**(10): p. 6131-6138.

10. Lin, Z., et al., *A high capacity small molecule quinone cathode for rechargeable aqueous zinc-organic batteries.* Nature Communications, 2021. **12**(1): p. 4424.

11. Zhao, Q., et al., *High-capacity aqueous zinc batteries using sustainable quinone electrodes.* Science advances, 2018. **4**(3): p. eaao1761.

12. Huang, J., et al., *Polyaniline-intercalated manganese dioxide nanolayers as a high-performance cathode material for an aqueous zinc-ion battery.* Nature communications, 2018. **9**(1): p. 2906.

13. Ma, L., et al., *Initiating a mild aqueous electrolyte Co 3 O 4/Zn battery with 2.2 V-high voltage and 5000-cycle lifespan by a Co (iii) rich-electrode.* Energy & Environmental Science, 2018. **11**(9): p. 2521-2530.

14. Wang, F., et al., *A rechargeable aqueous Zn 2+-battery with high power density and a long cycle-life.* Energy & Environmental Science, 2018. **11**(11): p. 3168-3175.

15. Häupler, B., et al., *Aqueous zinc-organic polymer battery with a high rate performance and long lifetime.* NPG Asia Materials, 2016. **8**(7): p. e283-e283.

16. Zhang, Y., et al., *Low-cost, resilient, and non-flammable rechargeable Fe-ion batteries with scalable fabrication and long cycle life.* Energy & Environmental Science, 2025. **18**(3): p. 1428-1439.
